# Altered Expression of Polycomb Group Genes in Glioblastoma Multiforme

**DOI:** 10.1371/journal.pone.0080970

**Published:** 2013-11-15

**Authors:** Gang Li, Charles Warden, Zhaoxia Zou, Josh Neman, Joseph S. Krueger, Alisha Jain, Rahul Jandial, Mike Chen

**Affiliations:** 1 Division of Neurosurgery, Department of Surgery, City of Hope National Medical Center, Duarte, California, United States of America; 2 Bioinformatics Core, Department of Molecular Medicine, City of Hope National Medical Center, Duarte, California, United States of America; 3 Flagship Biosciences, Boulder, Colorado, United States of America; University of Portsmouth, School of Pharmacy & Biomedical Sciences, United Kingdom

## Abstract

The Polycomb group (PcG) proteins play a critical role in histone mediated epigenetics which has been implicated in the malignant evolution of glioblastoma multiforme (GBM). By systematically interrogating The Cancer Genome Atlas (TCGA), we discovered widespread aberrant expression of the PcG members in GBM samples compared to normal brain. The most striking differences were upregulation of EZH2, PHF19, CBX8 and PHC2 and downregulation of CBX7, CBX6, EZH1 and RYBP. Interestingly, changes in EZH2, PHF19, CBX7, CBX6 and EZH1 occurred progressively as astrocytoma grade increased. We validated the aberrant expression of CBX6, CBX7, CBX8 and EZH2 in GBM cell lines by Western blotting and qRT-PCR, and further the aberrant expression of CBX6 in GBM tissue samples by immunohistochemical staining. To determine if there was functional significance to the diminished CBX6 levels in GBM, CBX6 was overexpressed in GBM cells resulting in decreased proliferative capacity. In conclusion, aberrant expression of PcG proteins in GBMs may play a role in the development or maintenance of the malignancy.

## Introduction

Glioblastoma multiforme (GBM) is an incurable primary brain tumor of the astrocytic lineage. Current therapies are modestly effective. Even with the most sophisticated treatment, median survival for patients with GBM is little more than a year [1]. To improve the ability to diagnose, treat, and prevent GBM, a better understanding of the molecular basis of this disease is necessary.

Recently, epigenetic aberrations have been implicated in the development of GBM [2], [3]. For example, in GBMs cancer specific concordant hyper-methylation of the CpG islands suppresses the promoters of hundreds of genes, giving rise to the “glioma-CpG island methylator phenotype (G-CIMP)” [4]. In addition to DNA methylation, another epigenetic mechanism involved in carcinogenesis appears to be malfunction or dysregulation of proteins that function in chromatin modification and remodeling. For example, EZH2 and BMI-1, both of which are reported to be involved in the maintenance and renewal of GBM stem cells, are also overexpressed in GBMs [5], [6]. EZH2 and BMI1 are members of Polycomb group (PcG) proteins. PcG family members are important epigenetic regulators that generally form protein complexes to perform their functions. The two main polycomb group complexes in mammals are Polycomb repressive complex 1 (PRC1), in which BMI-1 is a component and Polycomb repressive complex 2 (PRC2) in which EZH2 is an enzymatic component. PRC1 compacts chromatin and catalyses the monoubiquitination of histone H2A while PRC2 catalyses the methylation of histone H3 at lysine 27 to repress gene expression [7]-[9]. PcG mediated chromatin modification exerts a major influence on the maintaining of gene expression patterns of different cells that are set during early development and differentiation.

Given the complex interaction that occurs between PcG proteins, we hypothesized that large-scale dysregulation that is not confined to aberrant expression of EZH2 and BMI1 was needed for the development or maintenance of GBMs. To this end, we systematically interrogated The Cancer Genome Atlas (TCGA) database for expression of all of the PcG genes. In addition to previously reported alterations in EZH2 expression, we found remarkable differences in the expression of several PcG genes between GBMs and normal brain tissue. Of particular interest, our data revealed that expression of Chromobox 6 (CBX6), a component of PRC1, was suppressed in cancer specimens. Once this finding was validated, we demonstrated that CBX6 overexpression inhibited cell proliferation, suggesting that aberrant expression of CBX6 potentially promotes the development of GBMs.

## Materials and Methods

### Bioinformatics

#### Interrogation of The Cancer Genome Atlas (TCGA) database

TCGA data portal was accessed at: http://tcga-portal.nci.nih.gov/tcga-portal/AnomalySearch.jsp. Gene expression data from the Agilent G4502A_07 platform was used for initial analysis. A gene was considered overexpressed if the log_2_ Tumor/Normal Ratio was greater or equal (≥) to 0.5 or downregulated if the log_2_ Tumor/Normal Ratio was less or equal (≤) to -0.5. When “stringent criteria” were applied, a gene was considered overexpressed or downregulated if the log_2_ Tumor/Normal Ratio≥1 or≤-1 respectively.

#### Differential Gene Expression

Three studies [10]-[12] containing glioma samples of different grades and normal brain tissues were used to calculate differentially expressed genes that are up or down regulated in gliomas. P-values were calculated via t-test, and False Discovery Rates (FDR) were calculated based upon the distribution of t-test p-values using the method of Benjamini and Hochberg [13]. Genes were considered differentially expressed if they showed a fold-change greater than 1.2 with a FDR<0.05.

#### Visualization of Gene Expression for TCGA Subtypes

Robust multichip average (RMA) normalization [14] was applied to raw expression TCGA data (.CEL files for Affymetrix arrays) [15]. Molecular subtype labels from Verhaak et al. [16] were applied to the samples used in the training dataset for that study (N = 177). Expression was averaged among probes for genes with multiple probes on the Affymetrix HT HG-U133A array. The Partek Genomics Suite (Version 6.5) was used for normalization, hierarchical clustering, and dot-plots.

### Survival Analysis

Overall survival status and overall survival time was used to create survival plots for matched probes for PcG genes for 6 patient cohorts [10], [11], [15], [17]–[19]. Patients were divided into ‘high’ and ‘low’ expression groups based on median gene expression. Kaplan-Meier plot, log-rank test on the overall survival data was performed on this combined dataset. The p-value was calculated by log-rank test for the two-groups. 803 GBM patient samples were included in the survival analysis.

### Cell Lines

Human primary astrocytes derived from human cerebral cortex (Cell Applications Inc.) were cultured in Astrocyte Growth Medium (Cell Applications Inc.). T98G and U251MG glioblastoma multiforme cell lines were purchased from ATCC, and cultured in Dulbecco's Modified Eagle Medium supplemented with GlutaMAX and 10% fetal bovine serum (Hyclone).

### Constructs

Myc-DDK-tagged human CBX6 cDNA cloned into the pCMV6-Entry vector was purchased from OriGene. The pCMV6-Entry CBX6 construct was cut with EcoRI and re-ligated to serve as a control vector.

### Generation of Tetracycline-inducible CBX6 Overexpressing Cell Lines

U251MG cells were first stably transfected with two constructs, pGL4.51[luc2/CMV/Neo] (Promega) and pcDNA™6/TR (Life Technologies), the clone with the highest combined luciferase and Tet repressor expression was further stably transfected with a pcDNA™4/TO-CBX6 construct, the resulting clones were named as U251MG-Luc/TR-CBX6^TO^ (For detail, please see Figure S4).

### Antibodies

The following antibodies were used: CBX6 (#09-030, Millipore); CBX7 (#ab21873, Abcam); CBX8 (#09-031, Millipore); EZH2 (#5246S, Cell Signaling Technology) and GAPDH (#5174S, Cell Signaling Technology).

### Tissue Microarray

Brain glioblastoma multiforme tissue arrays (GL806a) were purchased from US Biomax Inc. Antigen retrieval was performed in 0.01M citrate buffer (pH 6.0) in a microwave oven. SuperPicture™ 3rd Gen Immunohistochemistry (IHC) Detection Kit (Invitrogen) was used for immunodetection of CBX6. The slides were scanned on an Aperio ScanScope by Flagship Biosciences (Westminster, CO.) and examined by two independent examiners. IHC staining was quantified using the H-score which was calculated by the formula: 3 X percentage of strongly staining nuclei + 2 X percentage of moderately staining nuclei + 1 X percentage of weakly staining nuclei, giving a range of 0 to 300.

### Quantitative RT PCR (qRT-PCR)

Total RNA was isolated using the Trizol reagent (Invitrogen). First-strand cDNA was synthesized using SuperScript III Reverse Transcriptase (Invitrogen) and oligo(dT)_20_. Quantitative PCR was performed in triplicate using SYBR green reagent (Bio-Rad) in the iQ5 machine (Bio-Rad). At least three independent experiments were performed for each assay. For CBX6, the sequences of the forward and reverse primers were 5′-AAACGGCGGATCCGAAAGGGAC-3′ and 5′-GCTGCAATGAGCCGCGAGTC-3′ respectively. For GAPDH, the sequences of the forward and reverse primers were 5′-AGGTGAAGGTCGGAGTCAAC-3′ and 5′-ATCTCGCTCCTGGAAGATGG-3′.

### Western Blots

Proteins were resolved using the Novex NuPAGE SDS-PAGE Gel System (Invitrogen) with 3-(N-morpholino) propanesulfonic acid (MOPS) running buffer and transferred to a nitrocellulose membrane (Bio-Rad). The enhanced Chemiluminescent (ECL) substrate from Pierce was used to detect horseradish peroxidase (HRP) activity from HRP linked secondary antibody (Cell Signaling Technology).

### Colony formation assay

pCMV6-Entry CBX6 and control constructs were transfected into U251MG cells using Lipofectamine® 2000 (Invitrogen), and then cultured in growth medium containing selection drug G418 (Sigma-Aldrich) at 1 mg/ml for three weeks. The colonies were fixed with 10% formalin, stained with 0.05% crystal violet (Sigma-Aldrich) and counted manually. Alternative quantification was also performed by measuring the absorbance at 540 nm after solubilizing cell-bound crystal violet using pure methanol.

### Statistical analysis

Statistical analysis was performed using GraphPad Prism (GraphPad Software, La Jolla, CA). Differences between the H-scores for CBX6 were analyzed by the Mann–Whitney U test. qRT-PCR data and data of the colony formation assay were analyzed by Student's t-test. For all tests, a P value<0.05 was considered to be significant.

## Results

### mRNA Expression of PcG genes in GBM

PcG gene products were initially identified as transcriptional repressors that when mutated resulted in dramatic changes of body patterning in Drosophila [20]. In mammals, PcG proteins form two major protein complexes, PRC1 and PRC2, to execute their functions. However, polycomb mediated gene silencing in mammals is complicated because there are usually multiple orthologs for each PcG protein (Table S1). Hence, to determine the role of PcGs in GBMs, it was necessary to comprehensively analyze TCGA data for aberrant expression of all of the PRC1 and PRC2 genes. We also examined several PRC2 associated genes such as DNMT3A, DNMT3B, SIRT1 and HDAC2.

**Figure 1:**
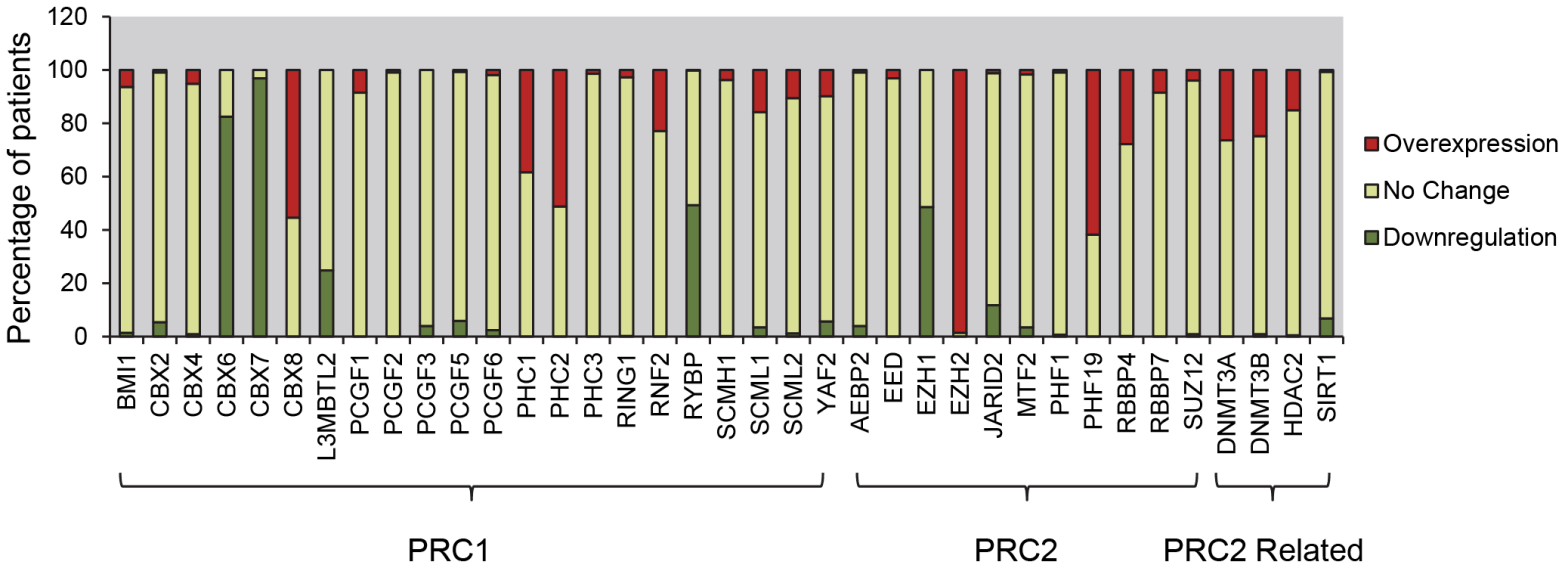
Interrogation of The Cancer Genome Atlas (TCGA) database of mRNA expression of PcG genes in glioblastoma multiforme (GBM). PcG gene expression statuses in glioblastoma patients were allocated into 3 different categories, overexpression (Tumor/Normal Ratio≥2, red); downregulation (Tumor/Normal Ratio≤0.5, green), and no change (0.5≤Tumor/Normal Ratio≤2, yellow). The stacked bar graph depicts the percentage of each category, calculated out of total 424 patients.

Stringent criteria were used to determine relative expression of a gene in GBMs (Tumor) to normal brain (Normal). Overexpression occurred when the Tumor/Normal Ratio≥2, whereas downregulation was indicated by a Tumor/Normal Ratio≤0.5. As shown in Figure 1, EZH2 overexpression was present in 98.6% of GBM samples, which is consistent with previous reports [6], [21]. Other PcG genes commonly overexpressed in GBMs include PHF19 (61.8%), CBX8 (55.4%) and PHC2 (51.2%). Contrary to previous studies [5], [22], overexpression of BMI1 in GBMs was not observed. PcG genes that were downregulated included CBX6 (82.5%), CBX7 (96.9%), RYBP (49.3%) and EZH1 (48.6%). We also performed less stringent analysis using Tumor/Normal Ratio≥1.4 and Tumor/Normal Ratio≤0.7 as criteria for overexpression and downregulation respectively. The results, which follow the same trend as the results of the high stringent analysis are provided as supplementary data (Figure S1).

To validate the dysregulation of these PcG genes in GBMs, we performed further analysis using three independent published data sets [10]–[12]. Consistent with the TCGA analysis, EZH2 was found to be significantly upregulated (6.4 to 12.9 fold changes) in GBMs samples of all three datasets. PHF19 and PHC2 were also up-regulated in the three datasets, whereas CBX8 was found to be upregulated in only one of the independent datasets. Likewise, it also revealed the consistent downregulation of CBX6, CBX7, RYBP and EZH1 in GBMs samples of all three datasets (Table 1 & 2). The confirmation of PcG dysregulation in independent datasets indicates the observed changes in PcG gene expression is not due to artifacts introduced by numerous confounding factors.

**Table 1:** PcG genes up-regulated in GBMs.

Cohort	Gravendeel (10)	Murat (11)	Sun (12)
	Fold Change (p value)	Fold Change (p value)	Fold Change (p value)
EZH2	12.9 (1.9×10^−6^)	11.7 (8.5×10^−4^)	6.4 (5.5×10^−13^)
PHF19	1.5 (3.7×10^−4^)	1.7 (0.0019)	1.7 (3.2×10^−4^)
CBX8	1.1 (0.01)	1.1 (0.036)	1.6 (5.0×10^−6^)
PHC2	1.7 (2.8×10^−5^)	1.8 (0.0020)	1.5 (7.2×10^−4^)

**Table 2:** PcG genes downregulated in GBMs.

Cohort	Gravendeel (10)	Murat (11)	Sun (12)
	Fold Change (p value)	Fold Change (p value)	Fold Change (p value)
CBX7	−4.7 (2.4×10^−6^)	−3.3 (8.5×10^−4^)	−2.7 (8.9×10^−11^)
RYBP	−2.0 (2.8×10^−5^)	−1.7 (0.0039)	−1.8 (2.5×10^−7^)
CBX6	−2.4 (3.5×10^−6^)	−1.8 (0.0010)	−1.8 (9.2×10^−11^)
EZH1	−1.6 (1.1×10^−5^)	−1.4 (0.0031)	−1.5 (9.1×10^−10^)

### PcG expression as a function of GBM subtype

**Figure 2:**
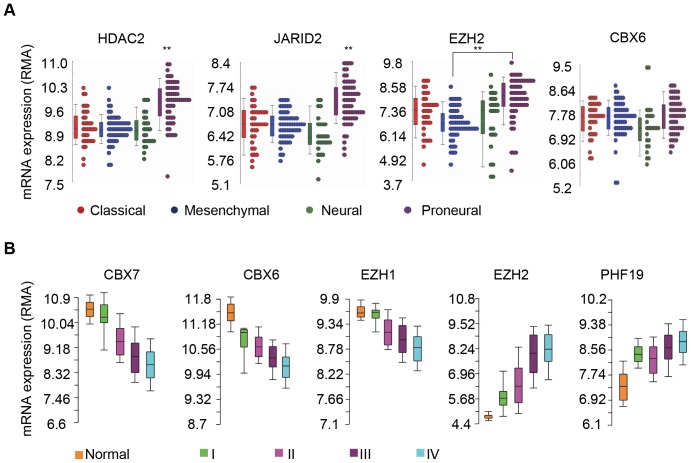
Expression of PcG genes in different glioblastoma subtypes and astrocytoma grades. (**A**) PcG gene expression in TCGA glioblastoma multiforme (GBM) subtypes. Using the TCGA data for GBM, pair-wise comparisons were conducted between all 4 of the molecular subtypes (Mesenchymal, Classical, Neural and Proneural, color coded). ** P<0.01. (**B**) Continuum of abnormal expression in PcG genes in different histological grades of astrocytomas. The Y-axis measures the background adjusted Robust Multi-array Average (RMA) which indicates the normalized expression level. Box-and-whisker plots show the distribution of mRNA expression in normal brain and different grades of astrocytomas (color coded). For detailed statistical analyses, please see Table S3 & S4.

While all GBMs share similar histological characteristics, e.g., presence of anaplastic glial cells, mitotic activity, vascular proliferation and necrosis, there is significant heterogeneity in the molecular profiles of GBMs amongst patients or even within an individual patient's tumor [23], [24]. Based on gene expression profiling, several subtypes of GBM have recently been identified. Verhaak et al. classified glioblastoma samples into four subtypes, i.e., classical, proneural, neural and mesenchymal subtypes using unsupervised hierarchical clustering analysis [16]. We examined the TCGA dataset to determine if PcG genes were differentially expressed in these GBM subtypes [15], [16]. Pair-wise comparisons revealed that the majority of the PcG genes were not differentially expressed in different subtypes of GBM (Table S2). Exceptions occurred with HDAC2 and JARID2, which were significantly upregulated in the proneural subtype, and with EZH2, which also had higher expression in proneural subtype but only when compared to the mesenchymal subtype (Figure 2A).

### Expression of PcG genes in different astrocytoma grades

GBMs (WHO Grade IV) are the most malignant form of astrocytoma. We speculated that there would be a continuum of PcG dysregulation as the astrocytoma became progressively more malignant. PcG gene expression was then examined in astrocytoma samples with different histological grades. Interrogation of a combined dataset [10]–[12] revealed that a grade-wise progression of PcG dysregulation was evident for select genes. CBX6, CBX7 and EZH1 shared a similar pattern and correlated negatively with increasing astrocytoma grade, whereas the opposite occurred with EZH2 and PHF19 (Figure 2B, table S3 and S4).

### Correlation between PcG expression and overall survival of GBM patients

Next, we examined a combined dataset including 6 GBM patient cohorts [10], [11], [15], [17]–[19] to determine if the PcG genes that were the most differentially expressed were related to GBM patient survival. We rationalized that genes with aberrant expression in GBM that also correlated with survival would be attractive candidates as therapeutic targets and/or biomarkers. Unfortunately, Kaplan-Meier survival analyses revealed that none of the dysregulated PcG gene significantly correlated with GBM patient survival (data not shown). However, in a cohort which includes low grade gliomas [10], significant survival benefits were observed for patients with low EZH2, PHF19, CBX8 and PHC2 expression, and high CBX7, CBX6, RYBP and EZH1 expression (Figure S2). Nonetheless, this benefit might only manifest those genes are differentially expressed between low and high grade gliomas.

### Expression of CBX6 is downregulated in GBM cell lines

**Figure 3:**
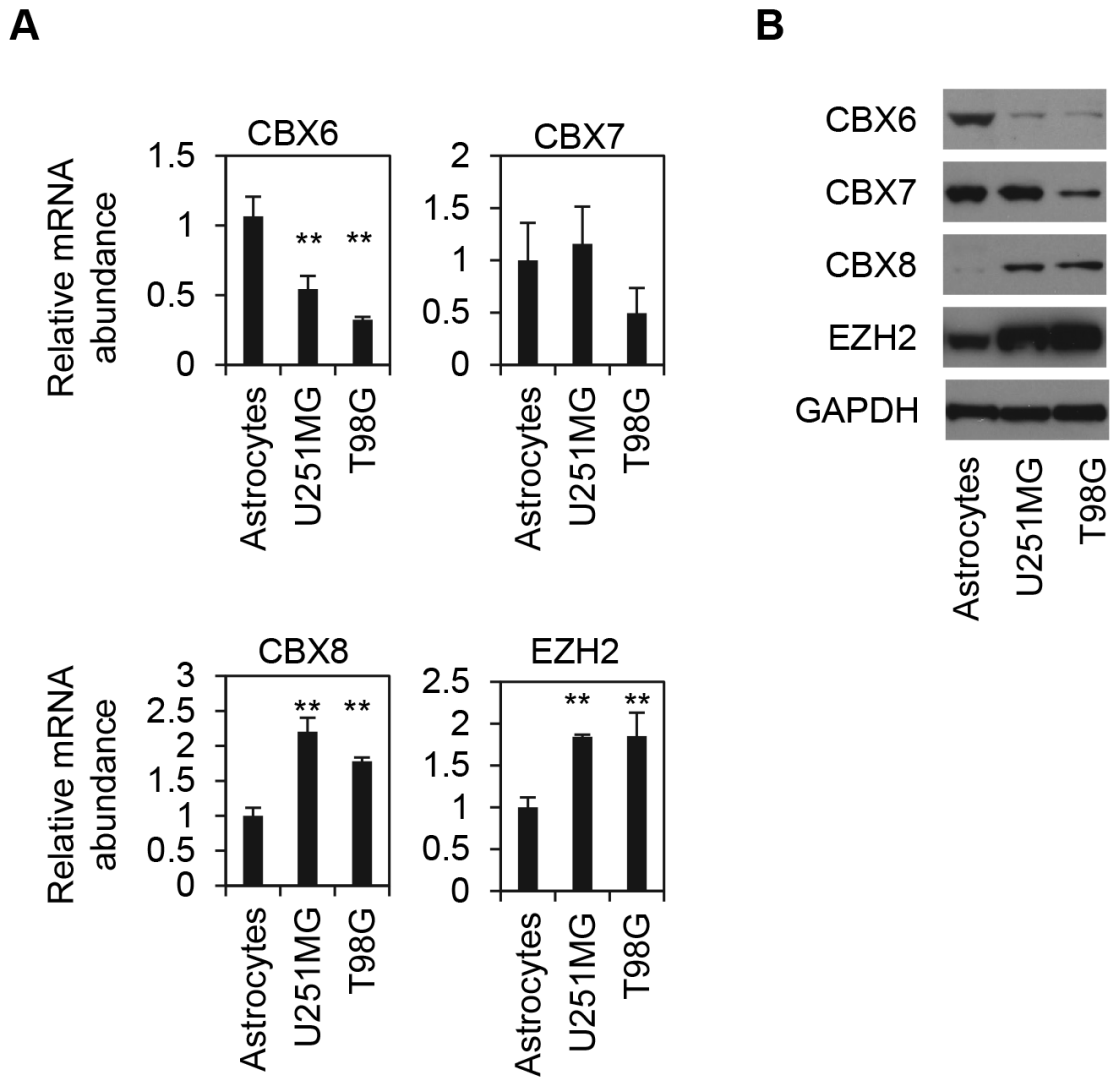
CBX6 expression is downregulated in human glioblastoma multiforme (GBM) cell Lines. (**A**) qRT-PCR analysis of the mRNA levels of multiple PcG genes in primary human astrocytes, U251MGand T98G human GBM cell lines. Expression was normalized to the GAPDH (Glyceraldehyde 3-phosphate dehydrogenase) gene and the average of each PcG expression in primary human astrocytes was arbitrarily set to one. Data represent the average of three independent experiments. Error bars represent standard deviation. ** (P<0.01). (**B**) Western blot analysis of the expression of multiple PcG proteins in primary human astrocytes, T98G and U251MG GBM cell lines. GAPDH was used as a loading control.

Out of the list of PcG genes that were analyzed in greater depth, we were most intrigued by the changes of the five Chromobox homolog genes (CBXs), which are the mammalian orthologs of Drosophila Polycomb (PC) gene. Through the chromodomain of CBX proteins, PRC1 complexes bind to the trimethylated lysine 27 of histone H3 (3mH3K27), the product of PRC2 catalysis, to find gene targets. As shown in Figure 1, the expression levels of CBX6 and CBX7 are downregulated, and the expression of CBX8 is upregulated in glioblastoma samples, whereas the expression of CBX2 and CBX4 remain unchanged. Cell lines provide a homogeneous population, and a strong positive correlation has been observed in gene expression patterns between cancer cell lines and primary tumors [25]. To extend the above observation, the expression of CBX6, CBX7, CBX8 and EZH2 in primary human astrocytes were compared with their expression in two GBM cell lines, T98G and U251MG. As shown in Figure 3, compared to their levels in primary astrocytes, expression of CBX6 is significantly downregulated, whereas the expression of CBX8 and EZH2 are upregulated at both the mRNA and protein levels in the GBM cell lines. On the other hand, CBX7 expression level was down-regulated in T98G, but not in U251MG cells glioblastoma cells, when compared to levels in primary astrocytes. Because of that CBX7 mRNA level is extremely low in normal primary astrocytes, approximately 20 times lower than the level of CBX6 (data not shown), we chose CBX6 for further studies.

### Expression of CBX6 is downregulated in clinical glioma samples

**Figure 4:**
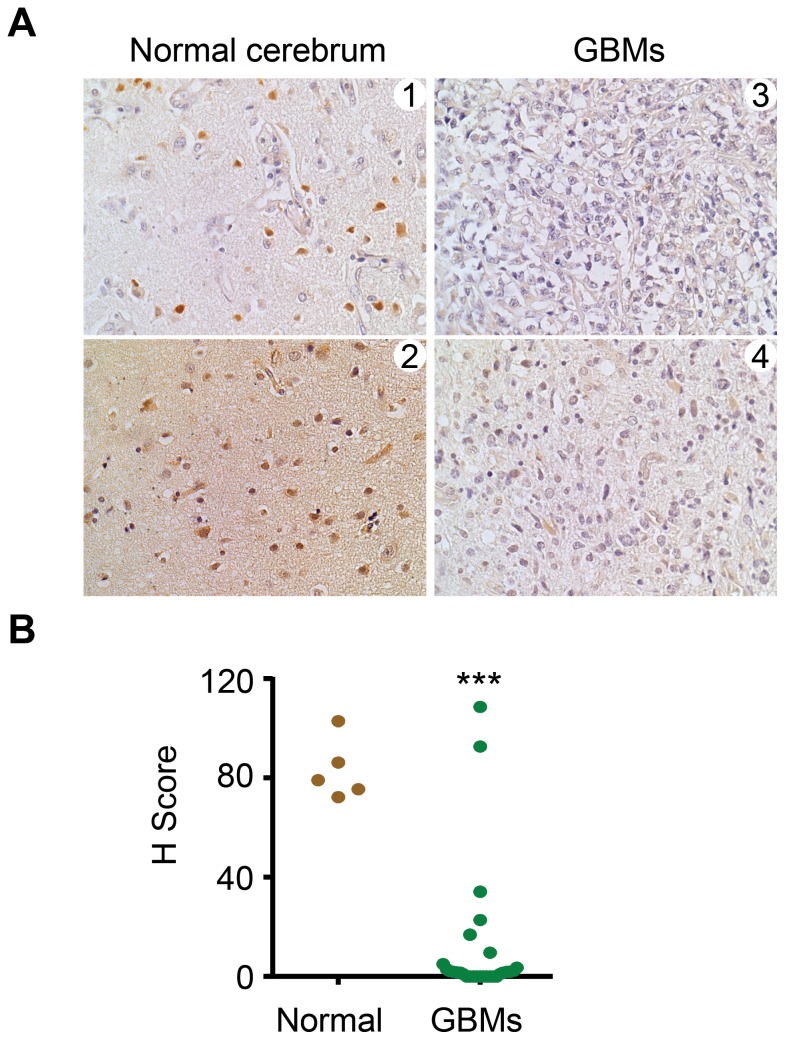
Aberrant expression of CBX6 in human glioblastoma multiforme (GBM). Human GBM tissue array was stained by immunohistochemistry using anti-CBX6 antibody. (**A**) Representative pictures of normal cerebrum (1, 2) and GBMs (3, 4) stained with CBX6 antibodies. (**B**) CBX6 levels were evaluated, H scores were calculated as in *Materials and Methods* and graphed. *** (P<0.001).

Next we analyzed CBX6 levels by immunohistochemistry using a tissue microarray containing 29 GBM cases and 5 samples of normal brain tissue (Figure 4A). To objectively describe the extent of CBX6 immunohistochemical staining, the degree of staining was quantified using the H score method (Figure 4B). All 5 samples of normal brain tissue were positively stained for CBX6 with a median H score of 83.2. Among the 29 GBM samples, 22 samples showed weak or no CBX6 staining with H score below 5, 5 samples showed modest staining with H score between 5 and 40 and 2 samples showed comparable staining to normal brain tissue (Figure 4B). These results confirmed that CBX6 expression is indeed downregulated in GBM samples, although some heterogeneity exists.

### Overexpression of CBX6 leads to cell growth arrest

**Figure 5:**
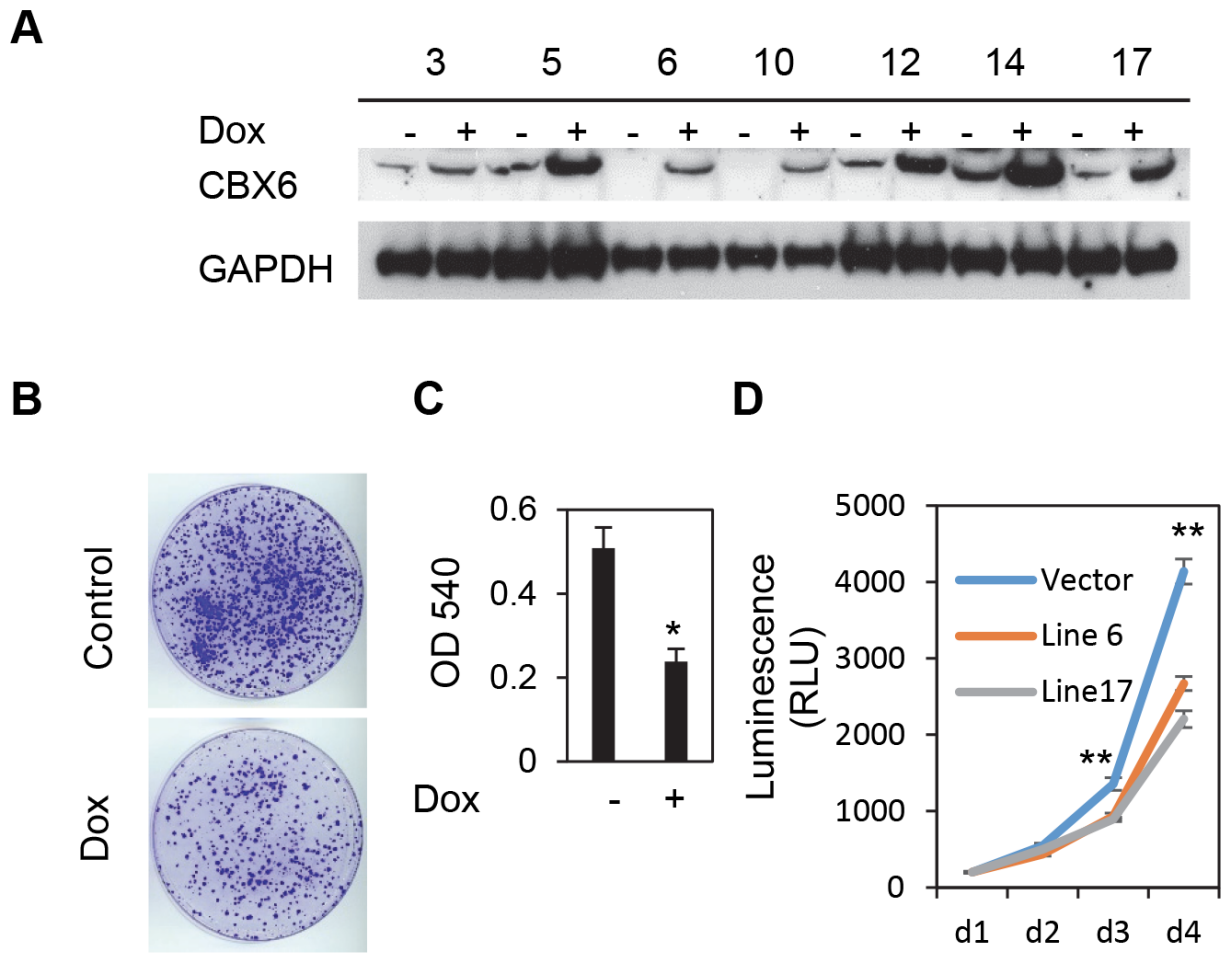
Overexpressing CBX6 gene inhibits the growth of U251MG cells. (**A**) Western blot analysis of the expression of CBX6 in U251MG-Luc/TR-CBX6^TO^ cell lines. 0.5ug/ml of Doxycycline was added to induce CBX6 expression. GAPDH was used as a loading control. Top labels indicate assigned number of each cell line. (**B**) Colony formation assay, U251MG-Luc/TR-CBX6^TO^ cells were treated with 0.5 µg/ml doxycycline to induce CBX6 expression. The colonies were stained with 0.05% crystal violet (CV). Shown is a representative of two independent experiments performed in triplicate with line 17. (**C**) Methanol was added to solubilize the crystal violet dye. Absorbance at 540 nm was read using DTX 880 plate reader (B.D.) Error bars represent standard deviation. * P<0.05. (**D)** U251MG-Luc-CBX6^TO^ cells (line 6 and 17) and U251MG-Luc-Vector^TO^ (vector control) cells were treated with doxycycline and cell proliferation was assessed using the CellTiter-Glo® Luminescent Cell Viability Assay (Promega). Shown is a representative of three independent experiments performed in quadruplicate. ** P<0.01.

To determine if CBX6 dysregulation has any functional ramifications as opposed to merely being a passenger alteration, we examined the effect of CBX6 overexpression on GBM cell proliferative capacity using a colony formation assay. U251MG cells were transfected with a construct encoding the CBX6 cDNA and a neo(R) cassette. After 21 days of G418 selection, the cells transfected with CBX6 formed significantly fewer and smaller colonies compared to the vector control (Figure S3A). Similar results were also obtained when using T98G glioblastoma cells (Figure S3B) indicating that CBX6 has an inhibitory effect on cell growth. To provide a well-controlled system to dissect the CBX6 function; we then established multiple tetracycline-inducible CBX6 overexpression stable cell lines based on U251MG (Figure 5A), and examined the effect of CBX6 overexpression on proliferative capacity using colony formation and ATP assays. U251MG-Luc/TR-CBX6^TO^ cells (line 17) were treated with doxycycline (+dox) to induce CBX6 overexpression. Compared to non-induced controls, the cells with induced CBX6 overexpression formed fewer colonies (Figure 5B). The cell proliferation inhibitory effect was also corroborated by measuring the absorbance after solubilizing cell-bound crystal violet (Figure 5C). Further, levels of ATP of U251MG-Luc/TR-CBX6^TO^ (+dox) cells (line 6 and 17) were dramatically decreased at days 3 and 4 compared to U251MG-Luc/TR-Vector^TO^ (vector control, +dox) cells, indicating cell proliferation was reduced upon overexpression of CBX6 (Figure 5D). The later control was included to isolate CBX6 effects from the potentially confounding effect of doxycycline on cell proliferation. Collectively, these data suggest that CBX6 has an inhibitory effect on cell growth in vitro.

## Discussion

PcG proteins have been shown to play a significant role in the epigenetic maintenance of cell identity. Unsurprisingly, recent studies strongly suggest that dysregulation of PcG proteins can influence the development or malignant evolution of cancers [26]–[28]. Indeed, PcG proteins EZH2, BMI1 and CBX7 have been shown to possess oncogenic or tumor suppressor functions in different tumors including GBMs [5], [6]. Compared with Drosophila, PcGs evolved towards greater complexity in mammals with approximately 28 family members [7]–[9]. Because many PcGs have orthologs that can substitute for each other in the functional complexes such as PRC1 and PRC2, we speculated that global imbalances maybe present because certain components were already known to be dysregulated.

A systematic bioinformatics analysis of PcG expression in GBMs was conducted using the TCGA database in this study. The survey revealed that EZH2, PHF19, CBX8 and PHC2 were the most frequently upregulated PcG genes; and CBX7, CBX6, EZH1 and RYBP were the most frequently downregulated PcG genes. The most frequently overexpressed PcG gene in GBMs was EZH2 (two fold increase in 98.6% GBMs). This finding is in agreement with previous reports [6], [21] and also consistent with the observed overexpression of EZH2 in many different types of solid tumors [29], [30]. EZH2 appears to be a promising therapeutic target in GBMs because of the extremely high frequency of overexpression and the observations that EZH2 inhibition severely retards the growth of cancer cells [6], [21].

Another discovery was that multiple Chromobox (CBX) genes had aberrant expression in GBMs. CBX proteins are mammalian homologues of the drosophila Polycomb (Pc) protein and components of mammalian PRC1 complexes. Mammalian genomes encode five homologs of drosophila Polycomb (Pc) gene: CBX2, CBX4, CBX6, CBX7 and CBX8. Binding of CBXs through their chromodomain to H3K27me is the classical model for PRC1 recruitment [9], although there are evidences supporting that H3K27me3/CBX-independent mechanisms can also recruit PRC1 complexes [31]–[34]. Though poorly understood, it has been suggested that each CBX protein has different functions [35]–[38]. Indeed, compared to normal brain, the expression of CBX2 and CBX4 does not change, CBX6 and CBX7 decreases, while the expression of CBX8 increases in GBMs indicating the five CBXs are differentially regulated. Both CBX7 and CBX8 have been reported to repress the INK4a/ARF tumor suppressor locus allowing normal cells to bypass senescence [39], [40]. Studies have not shown expression changes of CBX6 or CBX8 in tumors, whereas there are contradictory reports on changes of CBX7 expression. For example, CBX7 expression is upregulated in lymphomas and gastric tumors [41], [42], but downregulated in malignancies such as colon, thyroid, pancreatic, and urothelial carcinomas [43]–[46]. The discovery of CBX6 downregulation in GBMs is particularly intriguing, because virtually nothing is known about CBX6, and downregulation of CBX6 is marked and occurs consistently in independent GBM samples. Our data also showed that overexpression of CBX6 led to cell growth arrest, further studies are needed to address the mechanism behind this. Understanding the functional significance of the reciprocal expression pattern of CBX8 relative to CBX6 and CBX7 in GBMs might eventually leads to the development of compounds targeting the chromodomain of a specific CBX protein which potentially have anti-tumor efficacy.

Dozens of studies have used gene expression profiling to subtype gliomas to complement the morphological classification of gliomas [47]. GBM subtyping based on gene expression profiling represents a significant step forward towards the development of personalized treatment being tailored to the unique pattern of genetic changes in each patient's tumor. In this study we asked whether PcG genes are differentially expressed among the different subtypes of glioblastoma defined by TCGA [16]. The analysis revealed that the majority of the PcG genes are not differentially expressed in different subtypes of GBM. On the other hand, our study revealed that the histological grade of astrocytomas correlated with the degree of aberrant expression in a graded manner in many PcG genes. Among them, the expressions of EZH2 and PHF19 correlated positively with the astrocytoma grades, whereas the expressions of CBX7, CBX6 and EZH1 correlated negatively with astrocytoma grades. One interpretation of these findings was that these changes could occur due to active cell division since higher grades have greater proliferative capacity. Another, more intriguing possibility was that PcG expression changes were a cause or essential event for malignant progression.

In summary, emerging evidence indicates glioblastomas adopt changed epigenetic regulating machinery to maintain its malignancy as manifested here by altered expression of PcG proteins. In this study, we demonstrate that another member of the PcG family, CBX6, has a potential role in gliomagenesis. Specifically, we showed that expression of CBX6 is downregulated in glioblastoma cell lines and clinical samples and that induced CBX6 overexpression inhibited glioblastoma cell proliferation. Future studies will be required to assess its functional significance, elucidate its targets in the astrocytic genome, to corroborate the above findings using other in vitro and in vivo GBM model systems, and to understand why glioblastoma utilizes different CBXs during its malignant progression.

## Supporting Information

Figure S1 **Interrogation of The Cancer Genome Atlas (TCGA) database of mRNA expression of PcG genes in glioblastoma multiforme (GBM).** PcG gene expression statuses in glioblastoma patients were allocated into 3 different categories, overexpression (Tumor/Normal Ratio≥1.4, red); downregulation (Tumor/Normal Ratio≤0.7, green), and no change (0.7≤Tumor/Normal Ratio≤1.4, yellow). The stacked bar graph depicts the percentage of each category, calculated out of total 424 patients.
(PDF)

Figure S2 **Kaplan-Meier survival estimates overall survival of glioma patients according to the PcG expression.** A risk score was assigned to each patient which is a linear combination of the expression levels of the dysregulated PcG genes weighted by their respective upregulation or downregulation status. Specifically, the risk scores are calculated as follows: Risk score  =  EZH2 + PHF19 + CBX8 + PHC2 - CBX7 - CBX6 - RYBP - EZH1. Patients are divided into two groups based on median expression, and the Kaplan-Meier method was used to estimate overall survival time for the two groups. Statistical significance was analyzed using the two-sided log rank test. Median survival time for patients with high risk score (n  =  135) is 10.5 months, whereas median survival time for patients with low risk score (n  =  131) is 35.2 months, p  = 3.09e-08.
(PDF)

Figure S3 **Overexpressing CBX6 gene inhibits the growth of glioblastoma cells.** (**A**) & (**B**) U251MG (A) or T98G (B) cells were transfected with a vector expressing CBX6 cDNA, then put under drug (G418) selection for 21 days. The colonies were stained with 0.05% crystal violet. The empty vector pCMV6-Entry was used as a control. Shown is a representative of two independent experiments. (**C**) Number of colonies of U251MG cells were counted and graphed. Error bars represent standard deviation. * (P<0.05). Top panel, western blot analysis shows CBX6 is overexpressed in the transfected cells. (**D**) Methanol was added to solubilize the crystal violet dye. Absorbance at 540 nm was read using DTX 880 plate reader. Error bars represent standard deviation. * P<0.05. Top panel, western blot analysis shows CBX6 is overexpressed in the transfected cells.
(PDF)

Figure S4 **Diagrams of the vectors transfected into U251MG glioblastoma cell lines.** CMV, CMV promoter; Firefly (luc2) encodes firefly luciferase gene luc2; TetR, encodes Tet repressor gene; 2 x TetO2, two copies of the tet operator 2 (TetO2) sequence; Neor, Blastr and Zeocinr represent Neomycin, Blasticidin and Zeocin resistance gene cassettes respectively.
(PDF)

Table S1 List of Polycomb Group Proteins
(DOC)

Table S2 Differential expression of PcG genes in the four TCGA subtypes of GBM
(XLS)

Table S3 PcG genes differentially expressed in different grades of gliomas (Up-Regulated in high grade gliomas)
(DOC)

Table S4 PcG genes differentially expressed in different grades of gliomas (Down-Regulated in high grade gliomas)
(DOC)
