# Supplementary figures and images for: Altered Expression of Polycomb Group Genes in Glioblastoma Multiforme
Source: PLoS One. 2013 Nov 15;8(11):e80970. doi: 10.1371/journal.pone.0080970 (PMC3829908; doi:10.1371/journal.pone.0080970)

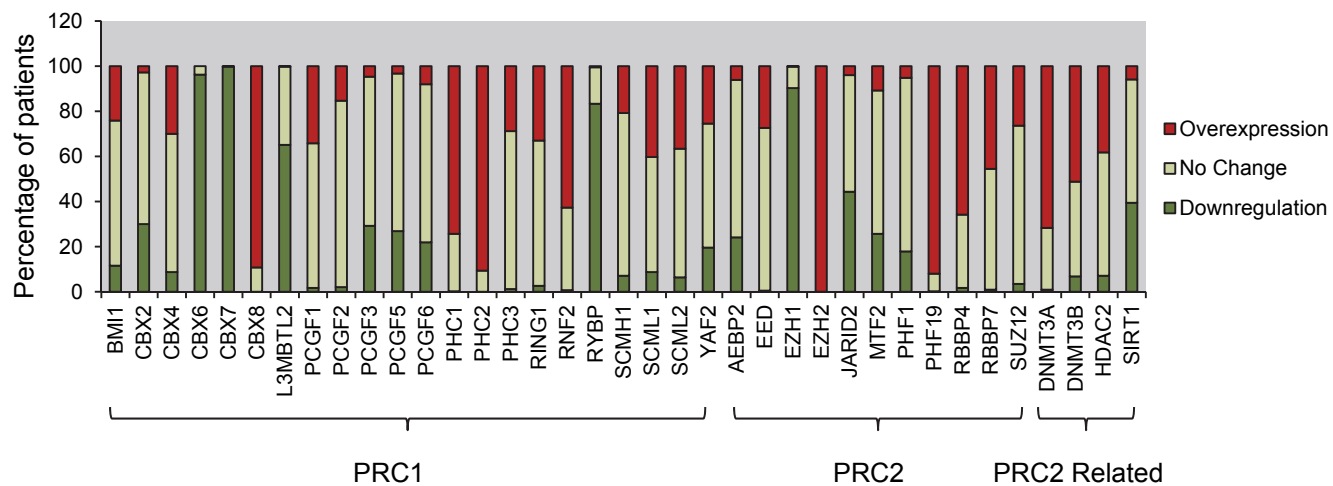

Li et al. Figure S1

Supplement: Figure S1 — Interrogation of The Cancer Genome Atlas (TCGA) database of mRNA expression of PcG genes in glioblastoma multiforme (GBM). PcG gene expression statuses in glioblastoma patients were allocated into 3 different categories, overexpression (Tumor/Normal Ratio≥1.4, red); downregulation (Tumor/Normal Ratio≤0.7, green), and no change (0.7≤Tumor/Normal Ratio≤1.4, yellow). The stacked bar graph depicts the percentage of each category, calculated out of total 424 patients. (PDF) [file pone.0080970.s001.pdf]

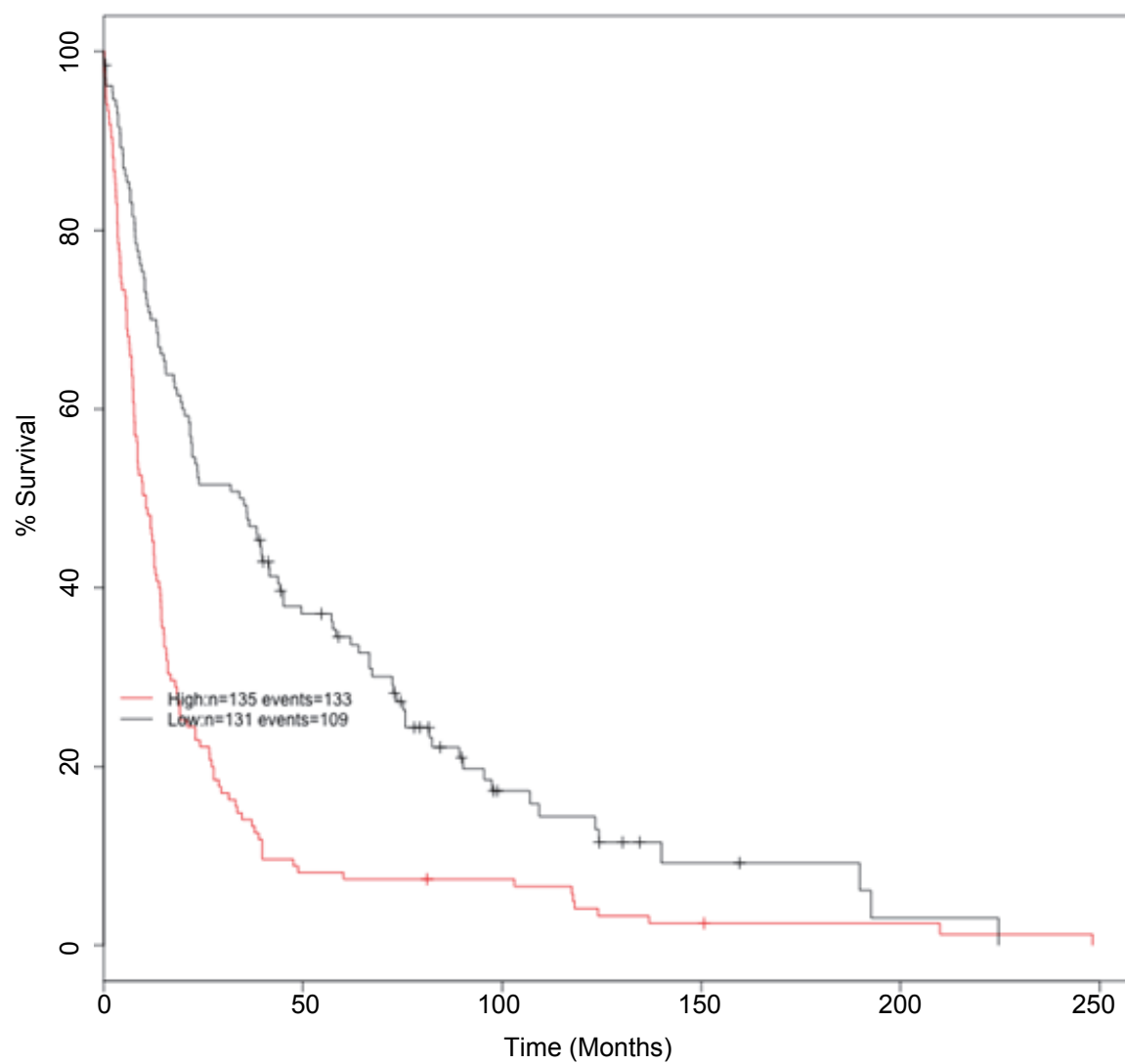

Li et al. Figure S2

Supplement: Figure S2 — Kaplan-Meier survival estimates overall survival of glioma patients according to the PcG expression. A risk score was assigned to each patient which is a linear combination of the expression levels of the dysregulated PcG genes weighted by their respective upregulation or downregulation status. Specifically, the risk scores are calculated as follows: Risk score = EZH2 + PHF19 + CBX8 + PHC2 - CBX7 - CBX6 - RYBP - EZH1. Patients are divided into two groups based on median expression, and the Kaplan-Meier method was used to estimate overall survival time for the two groups. Statistical significance was analyzed using the two-sided log rank test. Median survival time for patients with high risk score (n = 135) is 10.5 months, whereas median survival time for patients with low risk score (n = 131) is 35.2 months, p = 3.09e-08. (PDF) [file pone.0080970.s002.pdf]

**A**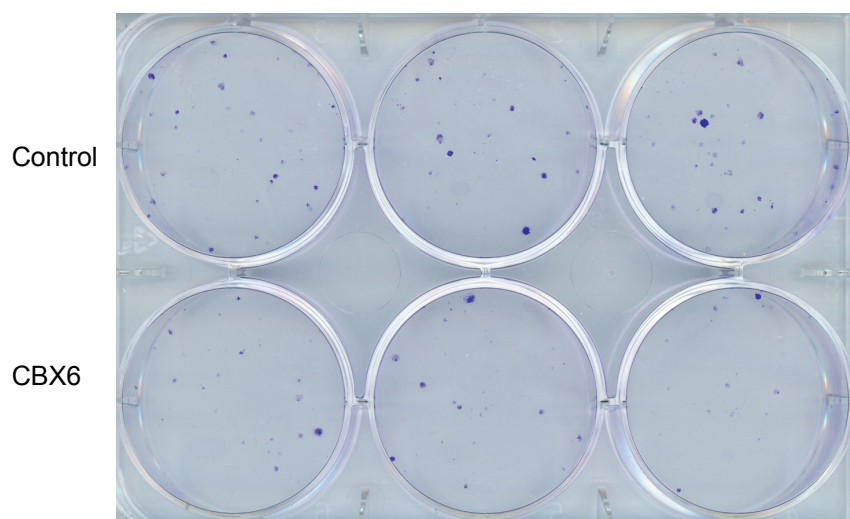**C**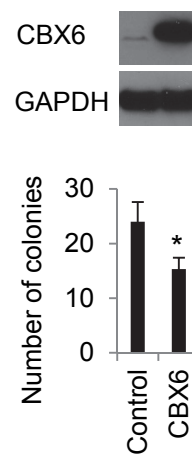**B**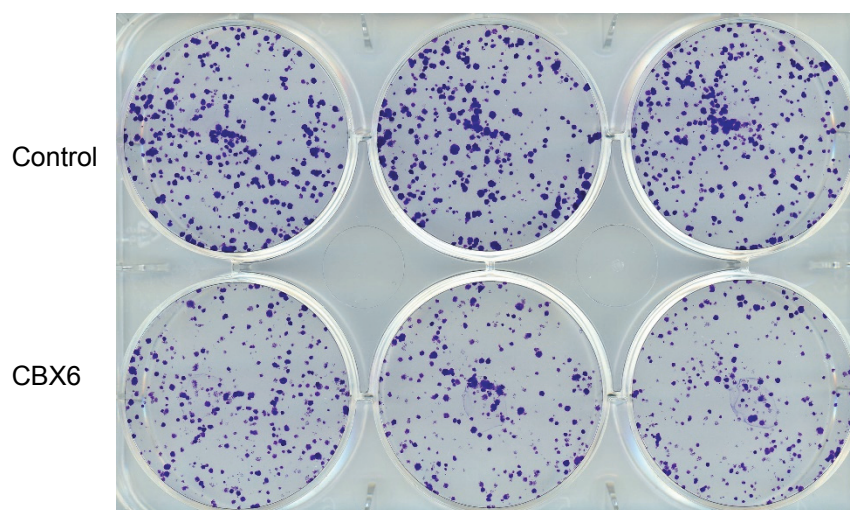**D**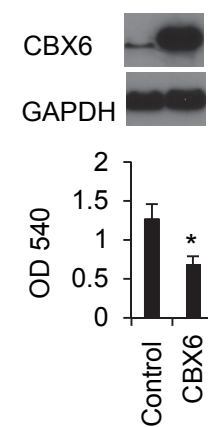

Li et al. Figure S3

Supplement: Figure S3 — Overexpressing CBX6 gene inhibits the growth of glioblastoma cells. (A) & (B) U251MG (A) or T98G (B) cells were transfected with a vector expressing CBX6 cDNA, then put under drug (G418) selection for 21 days. The colonies were stained with 0.05% crystal violet. The empty vector pCMV6-Entry was used as a control. Shown is a representative of two independent experiments. (C) Number of colonies of U251MG cells were counted and graphed. Error bars represent standard deviation. * (P<0.05). Top panel, western blot analysis shows CBX6 is overexpressed in the transfected cells. (D) Methanol was added to solubilize the crystal violet dye. Absorbance at 540 nm was read using DTX 880 plate reader. Error bars represent standard deviation. * P<0.05. Top panel, western blot analysis shows CBX6 is overexpressed in the transfected cells. (PDF) [file pone.0080970.s003.pdf]

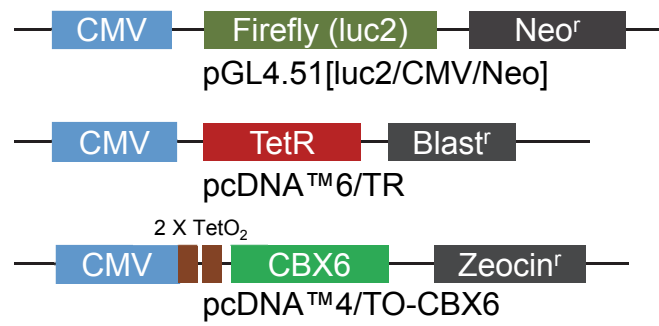

Li et al. Figure S4

Supplement: Figure S4 — Diagrams of the vectors transfected into U251MG glioblastoma cell lines. CMV, CMV promoter; Firefly (luc2) encodes firefly luciferase gene luc2; TetR, encodes Tet repressor gene; 2 x TetO2, two copies of the tet operator 2 (TetO2) sequence; Neor, Blastr and Zeocinr represent Neomycin, Blasticidin and Zeocin resistance gene cassettes respectively. (PDF) [file pone.0080970.s004.pdf]
