# Supplementary material for: Altered Expression of Polycomb Group Genes in Glioblastoma Multiforme
Source: PLoS One. 2013 Nov 15;8(11):e80970. doi: 10.1371/journal.pone.0080970 (PMC3829908; doi:10.1371/journal.pone.0080970)
Supplement: Table S1 — List of Polycomb Group Proteins (DOC) [file pone.0080970.s005.doc]

**Table S1**

| **List of Polycomb Group Proteins** | | |
| --- | --- | --- |
| Drosophila melanogaster | Homo sapiens | Other Aliases (Homo sapiens) |
| Polycomb Repressive Complex 2 (PRC2) | |  |
| E(z) | EZH1 | KMT6B |
|  | EZH2 | KMT6A |
| Esc | EED |  |
| Escl | ? |  |
| Su(z)12 | SUZ12 | JJAZ1 |
| Caf1 (Nurf55) | RBBP4 | RBAP48 |
|  | RBBP7 | RBAP46 |
| Pcl | PHF1 | PCL1 |
|  | MTF2 | PCL2 |
|  | PHF19 | PCL3 |
| Jarid2 | JARID2 | JMJ |
| Jing | AEBP2 |  |
| PRC2-Related | | |
|  | DNMT3A |  |
|  | DNMT3B |  |
| Sir2 | SIRT1 |  |
| Rpd3 | HDAC2 |  |
| Polycomb Repressive Complex 1 (PRC1) | | |
| Sce (dRing) | RING1 | RING1A, RNF1 |
|  | RNF2 | RING1B, RING2 |
| Pc | CBX2 | PC1 |
|  | CBX4 | PC2 |
|  | CBX6 |  |
|  | CBX7 |  |
|  | CBX8 | PC3 |
| Ph-p, Ph-d | PHC1 | HPH1 |
|  | PHC2 | HPH2 |
|  | PHC3 | HPH3 |
| Psc | BMI1 |  |
|  | PCGF1 | NSPC1 |
|  | PCGF2 | MEL-18 |
|  | PCGF3 |  |
|  | PCGF5 |  |
|  | PCGF6 | MBLR |
| Scm | SCMH1 |  |
|  | SCML1 |  |
|  | SCML2 |  |
| RYBP | RYBP |  |
|  | YAF2 |  |
| Sfmbt | L3MBTL2 |  |
| PhoRC complex |  |  |
| Pho | YY1 (?) |  |
| Phol | YY2 (?) |  |
| Sfmbt | L3MBTL2 |  |
|  | MBTD1 |  |
|  | SFMBT1 |  |
|  | SFMBT2 |  |
| L(3)mbt | L3MBTL |  |
|  | L3MBTL3 |  |
|  | L3MBTL4 |  |
| Polycomb repressive deubiquitinase (PR-DUB) | |  |
| Calypso | BAP1 |  |
| Asx | ASXL1 |  |
|  | ASXL2 |  |
|  | ASXL3 |  |
