# Supplementary material for: Altered Expression of Polycomb Group Genes in Glioblastoma Multiforme
Source: PLoS One. 2013 Nov 15;8(11):e80970. doi: 10.1371/journal.pone.0080970 (PMC3829908; doi:10.1371/journal.pone.0080970)
Supplement: Table S3 — PcG genes differentially expressed in different grades of gliomas (Up-Regulated in high grade gliomas) (DOC) [file pone.0080970.s007.doc]

**Table S3**

**PcG genes differentially expressed in different grades of gliomas**

(Up-Regulated in high grade gliomas)

|  | **High vs. Low*** | **IV vs III/II/I** | **IV vs III** |
| --- | --- | --- | --- |
| **AEBP2** | 1.2  (p=1.0 x 10^-4^) | 1.2  (p=1.5 x 10^-5^) | NS |
| **CBX2** | 1.4  (p=2.0 x 10^-4^) | 1.4  (p=3.4 x 10^-8^) | NS |
| **DNMT3A** | 1.4  (p=6.9 x 10^-6^) | 1.4  (p=3.9 x 10^-9^) | 1.4  (p=1.7 x 10^-29^) |
| **EZH2** | 3.5  (p=4.72 x 10^-18^) | 2.5  (p=1.6 x 10^-17^) | NS |
| **MTF2** | 1.3  (p=0.0013) | 1.3  (p=8.8 x 10^-6^) | NS |
| **PHC2** | NS | NS | 1.3  (p=2.7 x 10^-26^) |
| **PHF19** | 1.4  (p=8.2 x 10^-4^) | 1.4  (p=1.3 x 10^-5^) | 1.3  (p=3.1 x 10^-21^) |
| **RBBP4** | 1.3  (p=0.0060) | 1.2  (p=0.0013) | NS |
| **RYBP** | NS | 1.2  (p=0.0054) | 1.3  (p=1.6 x 10^-9^) |
| **SCMH1** | 1.3  (p=1.7 x 10^-6^) | 1.3  (p=2.0 x 10^-13^) | NS |
| **SUZ12** | 1.3  (p=1.3 x 10^-5^) | NS | NS |

High: Grade IV & III gliomas; Low: Grade I & II gliomas
