# Supplementary material for: Altered Expression of Polycomb Group Genes in Glioblastoma Multiforme
Source: PLoS One. 2013 Nov 15;8(11):e80970. doi: 10.1371/journal.pone.0080970 (PMC3829908; doi:10.1371/journal.pone.0080970)
Supplement: Table S4 — PcG genes differentially expressed in different grades of gliomas (Down-Regulated in high grade gliomas) (DOC) [file pone.0080970.s008.doc]

**Table S4**

**PcG genes differentially expressed in different grades of gliomas**

(Down-Regulated in high grade gliomas)

|  | **High vs. Low** | **4 vs 3/2/1** | **4 vs. 3** |
| --- | --- | --- | --- |
| **BMI1** | NS | -1.2  (p=7.2 x 10^-5^) | -1.2  (9.5 x 10^-7^) |
| **CBX6** | -1.3  (p=1.1 x 10^-5^) | -1.3  (p=1.1 x 10^-8^) | NS |
| **CBX7** | -2.1  (p=7.9 x 10^-14^) | -1.8  (p=4.2 x 10^-17^) | -1.2  (p=1.5 x 10^-43^) |
| **EZH1** | -1.4  (p=2.5 x 10^-8^) | -1.4  (p=6.4 x 10^-13^) | NS |
| **PCGF2** | NS | -1.4  (p=3.8 x 10^-9^) | -1.4  (p=3.3 x 10^-14^) |
| **PCGF5** | -1.5  (p=4.4 x 10^-5^) | -1.3  (p=2.8 x 10^-4^) | NS |
| **PCGF6** | NS | -1.2  (p=3.4 x 10^-7^) | -1.2  (p=6.1 x 10^-11^) |
| **PHC1** | NS | -1.2  (p=8.4 x 10^-5^) | -1.3  (p=8.3 x 10^-9^) |
| **SIRT1** | -1.3  (p=5.7 x 10^-4^) | -1.5  (p=5.8 x 10^-15^) | -1.5  (p=3.1 x 10^-28^) |
| **YAF2** | -1.2  (p=4.5 x 10^-4^) | -1.3  (p=9.4 x 10^-6^) | -1.2  (p=5.9 x 10^-6^) |

High: Grade IV & III gliomas; Low: Grade I & II gliomas
